# Associations of Health Literacy, Social Media Use, and Self-Efficacy With Health Information–Seeking Intentions Among Social Media Users in China: Cross-sectional Survey

**DOI:** 10.2196/19134

**Published:** 2021-02-25

**Authors:** Zhaomeng Niu, Jessica Willoughby, Rongting Zhou

**Affiliations:** 1 Section of Behavioral Sciences Division of Medical Oncology Rutgers Cancer Institute of New Jersey New Brunswick, NJ United States; 2 The Edward R Murrow College of Communication Washington State University Pullman, WA United States; 3 School of Humanities and Social Sciences University of Science and Technology of China Hefei China

**Keywords:** behavioral intention, health literacy, self-efficacy, social media

## Abstract

**Background:**

Empirical research has demonstrated that people frequently use social media for gathering and sharing online health information. Health literacy, social media use, and self-efficacy are important factors that may influence people’s health behaviors online.

**Objective:**

We aimed to examine the associations between health literacy, health-related social media use, self-efficacy, and health behavioral intentions online.

**Methods:**

We conducted a cross-sectional survey of adults 18 years and older (n=449) to examine predictors of health-related behavioral intentions online including health literacy, social media use, and self-efficacy in China using 2 moderated mediation models. Mediation and moderation analyses were conducted.

**Results:**

Self-efficacy mediated the effects of health literacy (B_indirect_=0.213, 95% CI 0.101 to 0.339) and social media use (B_indirect_=0.023, 95% CI 0.008 to 0.045) on health behavioral intentions on social media. Age moderated the effects of health literacy on self-efficacy (*P*=.03), while previous experience moderated the effects of social media use on self-efficacy (*P*<.001).

**Conclusions:**

Health literacy and health-related social media use influenced health behavioral intentions on social media via their prior effects on self-efficacy. The association between health literacy and self-efficacy was stronger among younger respondents, whereas the association between health-related social media use and self-efficacy was stronger among those who previously had positive experiences with health information on social media. Health practitioners should target self-efficacy among older populations and increase positive media experience related to health.

## Introduction

The remarkably fast growth of the internet has made it a major source for information sharing and acquisition. In the late 1990s, the internet became a main source for health information [1]. Advantages of using the internet for health information include (1) it is the most convenient and comprehensive source; (2) the information seekers remain anonymous; and (3) it helps reduce inequalities and eliminate barriers (eg, distance) [2]. A study [3] found that, in China, 76.3% of computer-based users and 68.8% of mobile-based (eg, smartphone) users sought health information on the internet [3]. Furthermore, the advent of social media has enabled more possibilities such as connecting people with similar health concerns or social support groups of patients [2,4].

With the rapid growth of social networking sites in China, the number of users and the variety of information on such sites have increased tremendously. As of 2018, there were 317 million active users of Weibo and 1 billion users of WeChat, which are the 2 main social media sites in China [5]. Weibo, a platform for microblogging, is often seen as the Chinese version of Twitter. WeChat is an instant messaging app that is similar to WhatsApp or Facebook Messenger, but it has more technological functions, such as free video or voice calls, group chat, public information sharing, mobile payments, and the ability to post pictures or videos [6].

Generally, extensive use of social media has been associated with informational or emotional need, professional development, social status, self-expression, and social interaction [7]. With an increasing awareness of health among the general public, a growing number of people in China are using social media for seeking and sharing health information [2,6]. Social media provide health information through multimedia affordances instead of solely text, which can increase the understanding of health information among populations with low health literacy [8]. Health literacy entails people's knowledge, motivation, and competency to access, understand, appraise, and apply health information in order to make judgments and decisions in everyday life concerning health care, disease prevention, and health promotion to maintain or improve their quality of life [9].

Additionally, a variety of health information and knowledge that used to be exclusive to health care providers are now available to health information seekers on social media [10]. Moreover, the user-generated nature of social media enables the sharing of health information and experiences, which provide views on health care from a patient’s perspective and increase patient empowerment [11]. Approximately, 40% of the individuals who sought health information on social media also shared their personal health experiences [12]. Furthermore, previous studies [13,14] have shown that use of social media has positive effects on health behaviors.

The health impact of social media in China has not been sufficiently studied and understood. For instance, research has focused on an examination of prominent health topics on social media [15] and how the general public views the impact of social media on health information acquisition [6]. Some studies have investigated the benefits and barriers [2], constructs of the theory of planned behaviors [16], cultural determinants, and doctor-patient communication [17] of health information intentions in China. Many studies in China have focused on health literacy among older adults or regionally [18-21]. However, no previous studies have comprehensively examined the potential relationships between health literacy, social media use, self-efficacy, and health behavioral intentions on social media in China based on the health literacy skills framework [22] and the social-cognitive perspective [23]. It is important to assess these constructs because studies have shown that health literacy levels in mainland China (the People's Republic of China excluding the special administrative regions of Hong Kong and Macau) remain low, which warrants further research regarding health literacy and related risk factors [24].

According to the health literacy skills framework, demographic factors, such as age, would moderate the development of health literacy while potential mediators, such as patient-provider communication [25] and knowledge [26], would mediate the effects of health literacy on health-related outcomes [22]. A number of studies [27-30] have found support for the mediating role of self-efficacy on health-related behavioral intentions [27,28]. Self-efficacy is the capacity to have positive effects on an individual’s health [29]. Lee et al [30] found that self-efficacy mediated the effects of health literacy on health behaviors.

It has also been documented that social media use plays a positive role in health behaviors [14]. Previous studies [16,31] in China also found the association between social media use and health behavioral intentions. In addition, previous research has identified the association between media use and self-efficacy [32]. Social-cognitive theory posits that one’s involvement with a subject grows over time through positive personal experiences, as one increases self-efficacy [33]. One study [34] found previous online health information seeking experience moderated seeking more of such information online. Therefore, health-related social media use and previous experience with using social media for health purposes could potentially influence self-efficacy and, consequently, impact health behavioral intentions.

**Figure 1:**
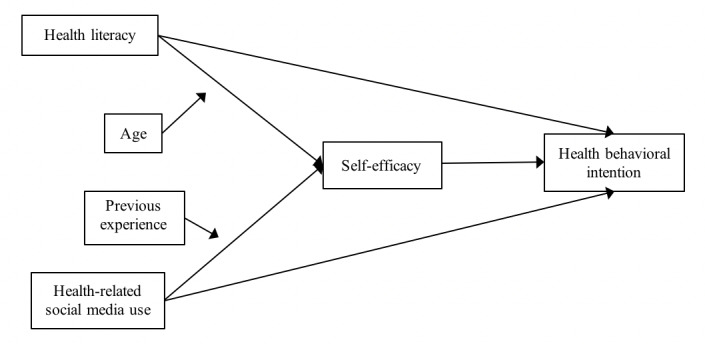
Conceptual framework.

The goal of this study was to examine the role of health literacy, social media use, and self-efficacy on health behavioral intentions on social media in China. Based on the literature, we proposed the following hypotheses (a conceptual model of the study is shown in Figure 1):

- Hypothesis 1: Self-efficacy will mediate the effects of health literacy on health behavioral intentions on social media.
- Hypothesis 2: Self-efficacy will mediate the effects of health-related social media use on health behavioral intentions on social media.
- Hypothesis 3: Age will moderate the effects of health literacy on self-efficacy.
- Hypothesis 4: Previous experience with using social media for health will moderate the effects of health-related social media use on self-efficacy.

## Methods

### Recruitment

We disseminated an online survey on Sina Weibo (Sina Corporation) in China using a paid advertisement service. Participants were required to be social media users and be at least 18 years of age. The first page of the survey was an online consent form including study information. After reading the consent form, indicating that they agreed to participate and were 18 years or older, respondents were allowed to proceed. After completing the survey, respondents were entered in a raffle to win CNY ¥300 (approximately US $42.86). The study was reviewed by the university institutional review board and was approved as an exempt study.

### Measures

Health-related social media use was measured by one question asking how frequently they have used social media for health information before on a scale ranging from 1 (never) to 7 (multiple times a day) (mean 4.01, SD 1.86). An English instructor at a Chinese university translated the questionnaire and used back-translation to ensure consistency in wording between English and Chinese versions of the survey.

Health literacy was measured by a scale adapted from Chinn and McCarthy [35] for health literacy measurement. This scale included seven 3-point items (rarely, sometimes, often), such as “When you talk to a doctor or nurse, do you give them all the information they need to help you?” and “Are you someone who likes to find out lots of different information about your health?” (mean 2.14, SD 0.40; Cronbach α=.71).

Self-efficacy was captured by the self-efficacy scale from Lee et al [29]. Five 7-point Likert-type items measured the degree of agreement with statements regarding self-efficacy in managing one’s health, such as “I have been able to meet the goals I set for myself to improve my health” and “I am confident I can have a positive effect on my health” (mean 5.04, SD 1.04; Cronbach α=.85).

Previous experience was measured by asking the respondents whether they found the health information on social media useful on three 7-point Likert scale items such as “In the past three months, health advice offered on social media sites has been useful to me” (mean 4.01, SD 1.58; Cronbach α=.91).

Behavioral intention was measured from an adapted multidimensional scale [36]. Three 7-point Likert-type items asked the extent to which respondents agree with the statements about their behavioral intention regarding health information on social media including “I will act upon the advice that is offered in the message in the near future,” “I will forward the message to my online acquaintances,” and “I will recommend the advice I read in the message to another person” (mean 3.84, SD 1.42; Cronbach α=.90).

We also measured demographic variables including age, sex, education, and family yearly income. Sex, education, and family yearly income were measured with 3 categorical questions while age was measured by asking participants to indicate their age in numbers (Multimedia Appendix 1).

### Statistical Analysis

To test the hypothesized associations, we used estimated direct and indirect effects in mediation and moderation models using SPSS statistical software (version 25.0, IBM Corp; PROCESS macro [37]). Mediation models (PROCESS model 4) were used for hypothesis 1 and hypothesis 2, whereas PROCESS model 7 was used to test the moderated mediation effect. Age, sex, education, and family yearly income were controlled as covariates for hypotheses 1, 2, and 4, whereas sex, education, and family yearly income were controlled as covariates for hypothesis 3.

## Results

### User Statistics

A total of 608 respondents began the questionnaire; however, 127 were removed due to declining to participate or incomplete participation (defined as more than 50% of the survey not completed), and 32 were excluded due to missing data. We had a final sample size of 449 (women: n=345; men: n=104; age: mean 25.23 years, SD 5.23, range 18-66). There were 242 participants aged from 23 to 30 years old (242/449, 53.9%). The education level of the sample was high, with 52.6% of the respondents (236/449) reporting having a bachelor’s degree, and 53.7% of the respondents (241/449) reported their family annual income was between ¥50,000 (approximately US $7150) to ¥200,000 (approximately US $28,600) (Table 1).

**Table 1:** Descriptive statistics.

Variable		Value
Behavioral intentions, mean (SD)		3.84 (1.42)
Health literacy, mean (SD)		2.16 (.42)
Self-efficacy, mean (SD)		5.04 (1.04)
Social media use, mean (SD)		3.80 (1.48)
Previous experience, mean (SD)		3.99 (1.60)
**Gender, n (%)**		
	Female	345 (76.8)
	Male	104 (23.2)
Age (years), mean (SD)		25.23 (5.23)
**Education, n (%)**		
	High school degree or lower	16 (3.6)
	College degree or some college	236 (52.6)
	Graduate degree or higher	197 (43.8)
**Family yearly income (CNY^a^), n (%)**		
	0-50,000	124 (27.6)
	50,001-100,000	112 (25.0)
	100,001-200,000	129 (28.7)
	>200,001	84 (18.7)

^a^An approximate exchange rate of 1 CNY to US $0.143 is applicable.

### Evaluation Outcomes

According to the results of model 4, self-efficacy mediated the effects of health literacy on health behavioral intentions on social media (*B*_indirect_=0.213, SE 0.060, 95% CI 0.101 to 0.339). Thus, hypothesis 1 was supported. The participants who had higher health literacy also had higher self-efficacy and then would be more likely to intend to perform health behaviors based on information acquired on social media (eg, use the health advice they found). Self-efficacy mediated the effects of health-related social media use on health behavioral intentions on social media (*B*_indirect_=0.023, SE 0.009, 95% CI 0.008 to 0.045), indicating hypothesis 2 was supported—the more the participants used social media for health, the higher their self-efficacy, and they would be more likely to have greater intentions to perform health behaviors on social media.

### Moderated Mediation

The results of moderated mediation models are shown in Table 2 and Table 3. According to the results of model 7, age moderated the effects of health literacy on self-efficacy (*B*=–0.041, SE 0.019, *P*=.03). The interaction had a negative effect on self-efficacy. Thus, hypothesis 3 was supported. The moderated mediation model accounted for 10.7% variance in health behavioral intention. Health literacy had a direct effect (*B*=0.345, SE 0.141, *P*=.02) as well as an indirect effect (Table 4) on health behavioral intention.

Previous experience with using social media for health moderated the effects of health-related social media use on self-efficacy (*B*=0.058, SE 0.015, *P*<.001). The interaction had a positive effect on self-efficacy. Therefore, hypothesis 4 was supported. This moderated mediation model accounted for 31.3% variance in health behavioral intention. Health-related social media use had a direct effect (*B*=0.315, SE 0.0309, *P*<.001) as well as an indirect effect (Table 5) on health behavioral intention.

**Table 2:** Regression results for effects of self-efficacy, age, and health literacy on health behavioral intention (*R*^2^=0.107, *P*<.001).

Variable	*B*^a^ (SE)	*P* value	95% CI
Sex	0.156 (0.152)	.30	–0.142 to 0.454
Education	0.214 (0.086)	.01	0.046 to 0.382
Family yearly income	0.141 (0.043)	.001	0.058 to 0.225
Health literacy	0.345 (0.141)	.01	0.068 to 0.621
Health literacy × age on self-efficacy	–0.041 (0.019)	.03	–0.077 to–0.004
Self-efficacy	0.318 (0.069)	<.001	0.183 to 0.453
Direct effect of health literacy on health behavioral intention	0.345 (0.141)	.01	0.068 to 0.621
Index of moderated mediation: age	–0.013 (0.006)	N/A^b^	–0.025 to –0.001

^a^Unstandardized final model coefficients.

^b^N/A: not applicable.

**Table 3:** Regression results for effects of self-efficacy, previous experience, and health-related social media use on health behavioral intention (*R*^2^=0.313, *P*<.001)

Variable	*B*^a^ (SE)	*P* value	95% CI
Age	0.052 (0.011)	<.001	0.029 to 0.074
Sex	0.138 (0.136)	.31	–0.128 to 0.405
Education	0.259 (0.078)	.001	0.106 to 0.412
Family yearly income	0.145 (0.037)	<.001	0.071 to 0.218
Health-related social media use	0.315 (0.031)	<.001	0.254 to 0.376
Health-related social media use × previous experience on self-efficacy	0.058 (0.015)	<.001	0.029 to 0.087
Self-efficacy	0.250 (0.057)	<.001	0.137 to 0.363
Direct effect of health-related social media use on health behavioral intention	0.315 (0.031)	<.001	0.254 to 0.376
Index of moderated mediation: previous experience	0.015 (0.005)	N/A^b^	0.006 to 0.026

^a^Unstandardized final model coefficients.

^b^N/A: not applicable.

**Table 4:** Conditional indirect effects of health literacy on health behavioral intentions by age.

Age (years)	Effect	SE	95% CI
20	0.294	0.079	0.145 to 0.452
25	0.226	0.062	0.109 to 0.351
30	0.158	0.059	0.061 to 0.290

**Table 5:** Conditional indirect effects of health-related social media use on health behavioral intentions by previous experience.

Previous experience (score)	Effect	SE	95% CI
mean – 1 SD (=2.414)	–0.017	0.012	–0.044 to 0.002
mean (=4.005)	0.006	0.007	0.009 to 0.020
mean + 1 SD (=5.597)	0.029	0.010	0.011 to 0.050

### Moderation Effects

Conditional indirect effects of health literacy on health behavioral intentions by age are shown in Table 4, and conditional indirect effects of health-related social media use on health behavioral intentions by previous experience are shown in Table 5. The positive indirect relationship between health literacy on health behavioral intentions was stronger among the younger segment of our sample (point estimate 0.294, SE 0.079, 95% CI 0.145 to 0.452). Additionally, the positive indirect effect of health-related social media use on health behavioral intentions was stronger among those participants who previously had positive experience with health information on social media, who were at one standard deviation above the mean (point estimate 0.029, SE 0.010, 95% CI 0.011 to 0.050). The moderated mediation results also revealed that self-efficacy remained a significant mediator no matter whether the participants were at 20, 25, or 30 years old. However, self-efficacy was only a significant mediator when the participants had the mean score of previous experience with health information on social media or one standard deviation above the mean score.

## Discussion

### Principal Findings

A substantial number of studies have examined health information on social media in China [6,16-18,28,38]; however, no previous studies have examined health behavioral intentions on a Chinese social media site from both the health literacy skills framework [22] or social-cognitive perspectives [23]. And there has been no study to comprehensively investigate how health information with different features on social media influenced the trust in such health information. Some studies [39,40] have examined the role of past experience and social media use individually, and some studies [18,20,21] have tested the associations between self-efficacy and other health outcomes; however, there are few empirical studies examining the relationship among health literacy, past experience, health-related social media use, and health behavioral intention on social media.

Our findings indicate that health literacy and health-related social media use influenced health behavioral intentions both directly and through their prior effects on self-efficacy in managing one’s health. Individuals with higher levels of health literacy had greater self-efficacy in managing their health and then, consequently, had greater health behavioral intentions on social media such as using the health information they found or sharing health information with others from the internet. Part of this finding is also consistent with those from previous studies [9,41,42] suggesting that health literacy is positively associated with information sharing. We also found that individuals who used social media for health purposes more frequently were more likely to report higher self-efficacy in managing their health and greater health behavioral intentions on social media. Social media usage for information could improve people’s psychological state and increase confidence and motivations to cope with uncertainties [43]. Therefore, people who use social media for health more frequently would be able to learn new information, cases, health experiences of others, and avoid potential risks, which could lead to a higher confidence in managing one’s own health. Our finding regarding the positive association between self-efficacy and health behavioral intentions is consistent with the social-cognitive theory perspective and previous empirical studies [44,45] examining effects of self-efficacy on different health behaviors and behavioral intentions.

Another important finding from our study pertains to the moderated mediation effects. Higher health literacy was associated with greater self-efficacy in health, which in turn was related to higher health behavioral intentions on social media. This relationship between health literacy and self-efficacy was moderated by age, suggesting that health literacy increased self-efficacy among younger social media users and eventually promoted their health behavioral intentions on social media. Younger social media users who had greater health literacy tended to have higher confidence in managing their own health and consequently had greater intentions to perform health behaviors. Among the older segment of social media users, no matter their health literacy level, their confidence in managing, improving, and generating positive effects on their health was lower than those of the younger groups. A number of studies [46-48] have found the negative association between age and self-efficacy. In one study [49], older adults who had lower incomes and lower education had relatively low self-efficacy, which was similar to our findings regarding the results of socioeconomic status and self-efficacy.

The positive relationship between health-related social media use and self-efficacy was stronger among those who had previously benefited from using social media for health. When people seek health information online, they usually not only experience increases in health knowledge but also find social support and help from people in similar situations [50]. Therefore, individuals with prior positive experiences using social media for health would have greater efficacy in exerting positive effects on one’s health. Previous experience with applying health advice found on social media in real life that resulted in good health results would improve their confidence in continuing to seek and use health information online.

### Implications

With the rapid growth of social media use, this study has important implications for health practitioners. A framework for health behavioral intentions was constructed based on the components of the health literacy skills framework [22] and the social-cognitive perspective [23].

Health literacy influenced self-efficacy and health behavioral intentions, which highlights the importance of health literacy level in China. The concept of health literacy is not popular in China and the quality of medical services provided in China varies significantly based on areas. This makes it important to improve the health literacy level in China so that people can have the ability to take effective and accurate actions related to health. Given the moderating role of age, participants between the ages of 25 and 30 years in our sample require more customized interventions, such as including carefully evaluated digital elements [51,52], to improve their self-efficacy in managing their health in health interventions.

Greater health-related use of social media was associated with higher self-efficacy and health behavioral intentions, indicating the importance of social media in understanding health behavioral intentions. Since the association between health-related social media use and self-efficacy was increased by positive experience with social media for health, health practitioners and scholars should aim to improve users’ experiences with social media regarding health information.

The findings are important for health scholars interested in understanding the factors that influence the intent to use health information on social media sites. This study also provides insights for health message designers who want to build effective health campaigns and distribute accurate and credible health information on social media platforms. Future studies should explicitly investigate how to improve health literacy levels and users’ experience with social media, such as by developing health literacy education programs [53].

### Limitations

Limitations in this study should be considered. First, we used a convenience sample on social media. The service used to advertise the survey link claimed to spread the survey post randomly, however, those who were interested in this study might share some similar traits (such as being in a younger population group or interested in this topic). Therefore, the sample was not truly representative of social media users in general, which might limit generalizability to other populations. Our sample was biased toward younger populations. Future studies could use different means to distribute the survey in order to reach a more diverse audience.

Second, while we asked people if they would act on the health information, we cannot verify or assess the potential accuracy of information that would be obtained. Future work should also consider credibility of the sources and information presented as part of the findings in terms of whether acting on information would be beneficial for health [54], especially in the online environment in which health misinformation may be rampant.

Finally, this study focused on investigating whether or not social media use could predict health-related behavioral intentions. Although previous research suggested that social media use could be influenced by cognition and behaviors [55], we did not test the reinforcing spiral framework of social media use in this study. This framework indicates that media use can influence attitudinal or behavioral outcomes, which can in turn affect habits of using media. According to this framework, media use can be an outcome of psychological processing and behaviors and also can influence psychological and behavioral results. Future studies should examine the reinforcing role of social media use in predicting health-related behavioral intentions on social media.

### Conclusions

Health literacy and health-related social media use in China participants influenced health behavioral intentions on social media via their prior effects on self-efficacy in health. The association between health literacy and self-efficacy was stronger among younger respondents, whereas the association between health-related social media use and self-efficacy was stronger among those who previously had positive prior experience with health information on social media. Our results provide insights for health practitioners and researchers and increase understanding of the mechanisms behind using social media for health. 

## Appendix

Multimedia Appendix 1 Variables.
