# Supplementary material for: Associations of Health Literacy, Social Media Use, and Self-Efficacy With Health Information–Seeking Intentions Among Social Media Users in China: Cross-sectional Survey
Source: J Med Internet Res. 2021 Feb 25;23(2):e19134. doi: 10.2196/19134 (PMC7952238; doi:10.2196/19134)
Supplement: Multimedia Appendix 1 [file jmir_v23i2e19134_app1.docx]

Table S1. Measures of variables of the study.

| Variable | Items | Mean (SD) |
| --- | --- | --- |
| Behavioral intentions | I will act upon the advice that is offered in the message in the near future. | 3.84 (1.42) |
|  | I will forward the message to my online acquaintances. |  |
|  | I will recommend the advice I read in the message to another person. |  |
| Health literacy | How often do you need someone to help you when you are given information to read by your doctor, nurse or pharmacist? | 2.14 (0.40) |
|  | Are you someone who likes to find out lots of different information about your health? |  |
|  | When you talk to a doctor or nurse, do you give them all the information they need to help you? |  |
|  | When you talk to a doctor or nurse, do you ask the questions you need to ask? |  |
|  | How often do you think carefully about whether health information makes sense in your particular situation? |  |
|  | How often do you try to work out whether information about your health can be trusted? |  |
|  | Are you the sort of person who might question your doctor or nurse's advice based on your own research? |  |
| Self-efficacy | I have been able to meet the goals I set for myself to improve my health. | 5.04 (1.04) |
|  | I am confident I can have a positive effect on my health. |  |
|  | I am actively working to improve my health. |  |
|  | I have set some definite goals to improve my health. |  |
|  | I feel that I am in control of how and what I learn about my health. |  |
| Social media use | How often do you use social media sites for health information? | 4.01 (1.86) |
| Previous experience | In the past three months, health advice offered on social media sites has been useful to me. | 4.01 (1.58) |
|  | In the past three months, health advice offered on social media sites help me a lot. |  |
|  | In the past three months, I followed health advice offered on social media sites. |  |
